# Microbial solutions to dietary stress: experimental evolution reveals host–microbiome interplay in *Drosophila melanogaster*

**DOI:** 10.1098/rspb.2024.2558

**Published:** 2025-03-26

**Authors:** Lucas Henry, Michael Fernandez, Andrew Webb, Julien Ayroles

**Affiliations:** ^1^ Ecology and Evolutionary Biology Department, Princeton University, Princeton, NJ, USA; ^2^ New York University, New York, NY, USA

**Keywords:** microbiome, dietary stress, experimental evolution, adaptive potential

## Abstract

Can the microbiome serve as a reservoir of adaptive potential for hosts? To address this question, we leveraged approximately 150 generations of experimental evolution in *Drosophila melanogaster* on a stressful, high-sugar diet. We performed a fully reciprocal transplant experiment using the control and high-sugar bacteria. If the microbiome confers benefits to hosts, then transplant recipients should gain fitness benefits compared with controls. Interestingly, we found that such benefits exist, but their magnitude depends on evolutionary history—mismatches between fly evolution and microbiome reduced fecundity and potentially exerted fitness costs, especially in the stressful high-sugar diet. The dominant high-sugar bacteria (*Acetobacter pasteurianus*) uniquely encoded several genes to enable uric acid degradation, mediating the toxic effects of uric acid accumulation due to the high-sugar diet for flies. Our study demonstrates that host genotype × microbiome × environment interactions have substantial effects on host phenotype, highlighting how host evolution and ecological context together shape the adaptive potential of the microbiome.

## 1. Introduction

The microbiome has profound effects on many aspects of organismal biology from modulating physiology [1] to providing pathogen protection [2] and influencing social interactions [3]. While the microbiome’s role as a key regulator of many host phenotypes is well established, its potential to influence host adaptation remains unclear. For hosts where microbes are tightly controlled and faithfully transmitted across generations, microbes can have a clear influence on host evolution [4–6]. For instance, the acquisition of nutrition-provisioning symbionts has enabled sap-feeding insects to expand into new ecological niches [7]. However, most eukaryotes acquire their microbiomes from the environment through weakly controlled transmission mechanisms [5]. This weak control disrupts the generational link between host and microbiome, potentially limiting microbial effects on host evolution [4,6,8]. Nevertheless, environmentally acquired microbes must survive and adapt to external environments, potentially developing beneficial traits that hosts could leverage. While this suggests microbiomes could influence host adaptation even without strict vertical transmission, the underlying mechanisms and extent of this influence remain poorly understood.

Environmentally acquired microbiomes appear to facilitate rapid host adaptation in several systems. For example, bean bugs acquire pesticide resistance from their soil-dwelling *Burkholderia* symbiont [9]. The gut microbiome of woodrats facilitates survival on toxic plant diets [10]. In plants, drought-adapted soil communities can increase fitness in drought conditions [11]. These studies suggest that microbes can adapt and evolve to stressful conditions in natural environments and buffer the stressor for the host, but much about the evolution of these interactions remains unclear—for example, we do not know to what extent these microbial effects depend on host genotypes. Host genetic variation may influence the responsiveness to microbial variation such that some combinations of host and microbiome differentially affect host phenotypes within species. The effects on host phenotype will also likely depend on the environmental context, where mismatches between the environment, host genotype and microbiome may negatively affect host fitness. In other words, host genotype × microbial genotype × environment (G_HOST_ × G_MICRO_ × E) interactions may shape how host–microbiome systems evolve. Such complex G_HOST_ × G_MICRO_ × E interactions are likely to be common, and yet their contributions in shaping host phenotypic variation and adaptation are underexplored.

The fruit fly, *Drosophila melanogaster*, has emerged as an excellent model to study host–microbe interactions, offering unique advantages for understanding microbiome dynamics and evolution. Flies have relatively simple microbiomes, largely environmentally acquired and often composed of fewer than 10 bacterial species from acetic acid and lactic acid bacteria families, yet these communities profoundly influence many different fly traits [12–14]. This simplicity, combined with the fact that many fly-associated microbes can be individually cultured and reconstituted in various combinations to inoculate sterile flies [14,15], provides unprecedented experimental control over microbiome composition. Indeed, recent work in *Drosophila* has provided important insights into how microbiome interactions shape host adaptation and fitness. Martino *et al*. [16] demonstrated that bacterial adaptation to host diet is a key force shaping the *Drosophila–Lactobacillus* association, with mutations arising in bacterial metabolic pathways that enhanced both bacterial fitness and host growth promotion. Additionally, Gould *et al*. [17] showed that interactions between different members of the microbiome significantly impact host fitness in *Drosophila*, with certain combinations of gut bacteria providing greater benefits than others. These studies highlight how both bacterial adaptation and microbe–microbe interactions contribute to host phenotypes. However, these studies focused on a single host genetic background and thus provide limited insight into G_HOST_ × G_MICRO_ interactions. Furthermore, the fly gut environment closely resembles that of other animals, featuring similar metabolic pathways, immune responses and host–microbe signalling mechanisms. These parallels make fly microbiome research broadly relevant to other systems. With both the feasibility of microbiome manipulations and rich genetic resources, including powerful tools for genome manipulation and thousands of characterized genetic variants, *Drosophila* represents an ideal model to study the interplay between host genetics, microbiome and environment (i.e. G_HOST_ × G_MICRO_ × E interactions) in shaping host phenotypes and adaptation.

Experimental evolution has provided transformative insights into adaptive processes, revealing mechanisms of natural selection that would be impossible to study through comparative approaches alone [6,18,19]. Within this field, the Evolve and Resequence (E&R) framework has proven particularly powerful for measuring genomic responses to selection [20]. This approach combines the controlled conditions of laboratory evolution with modern genomic tools, allowing researchers to track adaptive changes at unprecedented resolution. Although E&R experiments traditionally focused on the organism targeted by selection (e.g. yeast, flies, mice), recent work has revealed that the E&R approach can also identify changes in the microbiome associated with evolved populations [19]. While these parallel changes in host and microbiome are intriguing, demonstrating their adaptive significance requires direct experimental testing. To determine whether observed changes in the microbiome over generations of selection actually contribute to host adaptation, experimental validation through microbiome transplants between control and evolved populations is essential.

**Figure 1.**
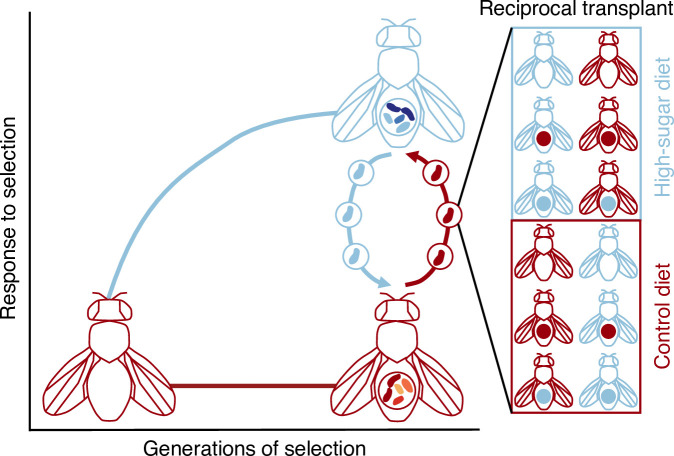
Leveraging approximately 150 generations of fly adaptation to the high-sugar (HS) diet to study the adaptive potential of the microbiome. The conceptualized evolutionary trajectory shows control (C) flies and microbiome in red and HS flies and microbiome in blue. After approximately 150 generations of selection, we performed a fully reciprocal host × microbiome × diet transplant experiment for all combinations of fly genotypes (C or HS), microbes (sterile, C or HS) and diets (C or HS). Together, this approach allows for the assessment of the benefit of adapted relative to non-adapted microbes in both host genetic and environmental contexts.

Here, we leverage approximately 150 generations of experimental evolution where flies and microbes were exposed to a nutritionally stressful, high-sugar (HS) diet. HS diets exert stress on flies, and flies exhibit similar obesity-like phenotypes as humans with elevated triglycerides, insulin resistance and shortened lifespans [21–24]. Dietary stressors also reshape the microbiome [14,25], which may also impact how flies evolve in the HS diet. Our goal was to first determine how HS selection changed the microbiome. Then, we hypothesized that transplanting the adapted microbiome into a non-adapted fly genotype would confer adaptive traits on the stressful HS diet. Through a fully reciprocal host × microbiome × diet transplant experiment (figure 1), we evaluate how each component contributes to fecundity in flies.

## 2. Material and methods

### (a) Fly populations

All fly populations were maintained at 25°C with 12 h light : dark cycles. The base ancestral population was derived by round-robin crossing a subset of the Global Diversity lines [26], which were then maintained at large population size (>10 000 flies) and allowed to interbreed freely for approximately 50 generations before HS selection began. The control (C) diet was composed of 8% glucose (Fisher Scientific D16−10), 8% brewer's yeast (MPI Biomedicals IC90331225), 1.2% agar (Fisher Scientific BP1423−2), with 0.04% phosphoric acid (Fisher Scientific A242SK−212) and 0.4% propionic acid (Fisher Scientific AC149300025) as preservatives. Three replicate populations for HS selection were derived from the base population and maintained on the same diet, but with an additional 12% sucrose (MP Biomedicals 902978) and at a lower population size (>5000 flies/cage). The control population was the ancestral population maintained at a large population size (>10 000 flies) in parallel on the C diet during the entirety of the HS selection. The approach used here allowed for natural selection on HS diets; we did not guide or select for particular traits, which is a common approach in experimental evolution studies in *Drosophila*. We consider the two populations, C and HS, to be two different genotypes, though note they are outbred and retain genetic diversity that is unique to the C or HS selection regime [27].

### (b) Identifying the microbiome response to selection in the high-sugar diet

We first profiled the microbiomes from single, age-matched adults (7–10 days post-eclosion) from both C and HS populations using 16S rRNA amplicon sequencing. In brief, DNA was extracted using the Zymo Quick-DNA extraction kit (Zymo D3012). The 16S V1-V2 region was amplified, pooled and digested with BstZ17I (NEB R3954) to deplete *Wolbachia* reads [28]. Libraries were sequenced using 300 bp paired-end reads using the Illumina MiSeq platform at the Princeton University Genomics Core. We used QIIME2 v.2019.4 [29] to process reads, cluster into amplicon sequence variants (ASVs) with DADA2 [30] and assign taxonomy with Greengenes classifier [31] trimmed to the 16S rRNA V1-V2 regions. Data was imported into phyloseq for visualization [32]. Flies with fewer than 500 reads/individual were discarded before the analyses. To identify differentially abundant bacteria between the C and HS flies, we used analysis of composition of microbiomes (ANCOM) [33] implemented in QIIME2. We then isolated the most differentially abundant bacterium from HS flies, *Acetobacter pasteurianus*, and the significantly differentially abundant bacterium within the *Acetobacter* genus and enriched in C flies, *Acetobacter indonesiensis*. These two strains were used in the microbiome transplant. For simplicity, we refer to *A. indonesiensis* as C *Acetobacter*. We note several *A. pasteurianus* strains have recently been reclassified into *Acetobacter oryzifermentans* [34], but for simplicity, we refer to this strain as HS *Acetobacter*.

To further characterize the mechanisms underlying microbial adaptation to the HS diet, we performed whole genome sequencing on the C and HS *Acetobacter* strains. Libraries were prepared following the manufacturer's instructions using the Illumina PCR-free Library Prep kit, and then 150 bp paired-end reads were sequenced on an Illumina NovaSeq platform at the Princeton University Genomics Core. Genomes were assembled using SPAdes [35] with isolate flag and annotated using the Rapid Annotation using Subsystem Technology (RAST) server [36]. We compared average nucleotide identity (ANI) between the C and HS *Acetobacter* using OrthoANIu [37]. Then, to identify functional changes, we manually compared the RAST annotations to identify pathways unique to either C or HS *Acetobacter*. We also generated the RAST annotations from two other abundant bacteria in the C microbiome and compared them with the HS *Acetobacter* annotations: *Algoriella xinjiangensis* (National Centre for Biotechnology Information (NCBI) accession FOUZ01000040.1) and *Acetobacter persici* (NCBI accession JOPC00000000.1). To confirm the differences between C and HS *Acetobacter* strains, we identified orthologues between the two *Acetobacter* genomes using OrthoFinder v2.5.2 [38]. Then, to gain insight into the function of the putative genes, we performed BLASTP v.2.10.1+searches against the NCBI non-redundant protein sequence database [39].

The genomic analysis performed above suggested that the HS *Acetobacter* genome encoded several pathways for urea degradation, but were not present in the C *Acetobacter* genome. To validate this observation, we measured the ability of the *Acetobacter* strains to degrade uric acid in fly food. Uric acid was added at 50 µmol concentration to 30 µl of C and HS diets. Then, 10 µl of C or HS *Acetobacter* (or sterile PBS control), normalized to OD_600_ = 0.1 (approximately 10^7^ cells ml^−1^ [15]), was added to the diet and incubated for 4 days at 25°C to degrade the uric acid. Uric acid concentration was measured using the Amplex Red Uric Acid/Uricase kit (ThermoFisher A22181) following the manufacturer's instructions. ANOVA was used to analyse the effects of bacteria (C or HS *Acetobacter*) and diet on the uric acid degradation by bacteria, with *post hoc t*-tests with Bonferroni correction for the mean difference in uric acid degradation between diets for each microbe treatment.

### (c) Transplanting microbiomes to test for adaptive significance

To investigate the impact of host genetic, microbial and environmental interactions on host fitness, we performed a fully reciprocal, host × microbiome × environment transplant (figure 1) using the C and HS *Acetobacter* strains. *Acetobacter* strains were cultured in liquid deMan Rogosa Sharpe (MRS, Sigma Aldrich 1106610500), density was normalized to OD_600_ = 0.1 (approximately 10^7^ cells ml^−1^) and 50 µl of bacteria (or phosphate-buffered saline (PBS) for sterile treatments) was used to inoculate axenic eggs in sterile diets [15]. For each treatment, 4−12 replicate populations were maintained. Flies were then reared in a biosafety cabinet at 25°C and 12 : 12 h light : dark cycle through the lifespan of the recipient flies. Success of the transplant (and maintenance of the sterile treatments) was confirmed by screening on MRS media (electronic supplementary material, figure 2).

**Figure 2.**
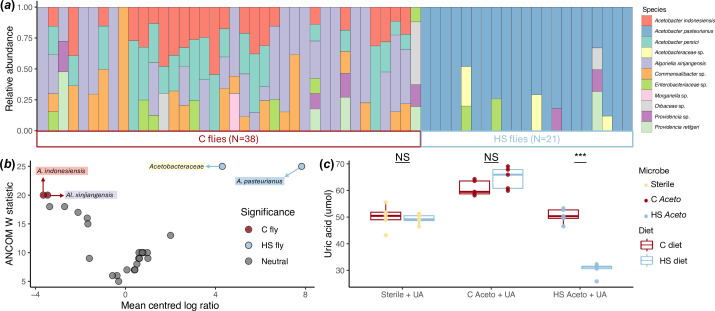
The microbiome in HS flies is different from C flies. (*a*) Relative abundance of bacteria between individual flies (*n* = 38 C flies, 21 HS flies). (*b*) ANCOM results for differentially abundant bacteria between C and HS flies. Each point represents an ASV. Colour denotes significance, i.e. whether the ASVs were significantly enriched in C (red) or HS (blue) flies. Labels denote taxonomic classification, coloured to match relative abundance in 2*a*. (*c*) Uric acid degradation by HS *Acetobacter* in the *in vitro* diet assay. Boxplots are coloured by diet with points coloured by *Acetobacter* treatment. Significance using *post hoc t*-tests with Bonferroni correction is marked above the box plots (NS = not significant, *** = p < 0.0001).

We then measured fecundity at a single time point in the recipients (*n* = 664 total, electronic supplementary material, table 1 for sample sizes). Females (8–10 days post-eclosion) were placed in a 24 well plate with oviposition media. Oviposition media was the same as the fly diet, but with yeast extract (Sigma Aldrich Y1625), which makes the diet transparent and easier to count eggs. Females laid eggs on both C and HS media. Fecundity from all individuals was initially measured in both media, but media did not affect egg laying (electronic supplementary material, figure 3, Mann–Whitney–Wilcoxon test, W = 50 964, *p* = 0.09), and data were pooled from both oviposition media for analysis. To minimize plate effects, two treatments were assayed on each plate, and treatments were randomly assigned across plates. Females laid eggs for approximately 4–6 h at 25°C during the late afternoon (14.00−20.00) to mimic the same time span for fly population maintenance during selection. We used a zero-inflated Poisson distribution implemented in glmmTMB [40], testing for the effects of interactions between fly genotype, *Acetobacter* strain and diet with oviposition plate (*n* = 29 plates) as the random effect. Model selection between models with and without interactions was performed using a likelihood ratio test. *Post hoc* tests on the fitted model were performed using emmeans [41] with Tukey corrections for multiple comparisons. To test the contribution of the different terms (i.e. fly genotype, microbe, diet and their interactions), we sequentially added each term and interactions and then fitted the model. We estimated the marginal R^2^ for each model using the delta method in MuMIn [42].

**Figure 3.**
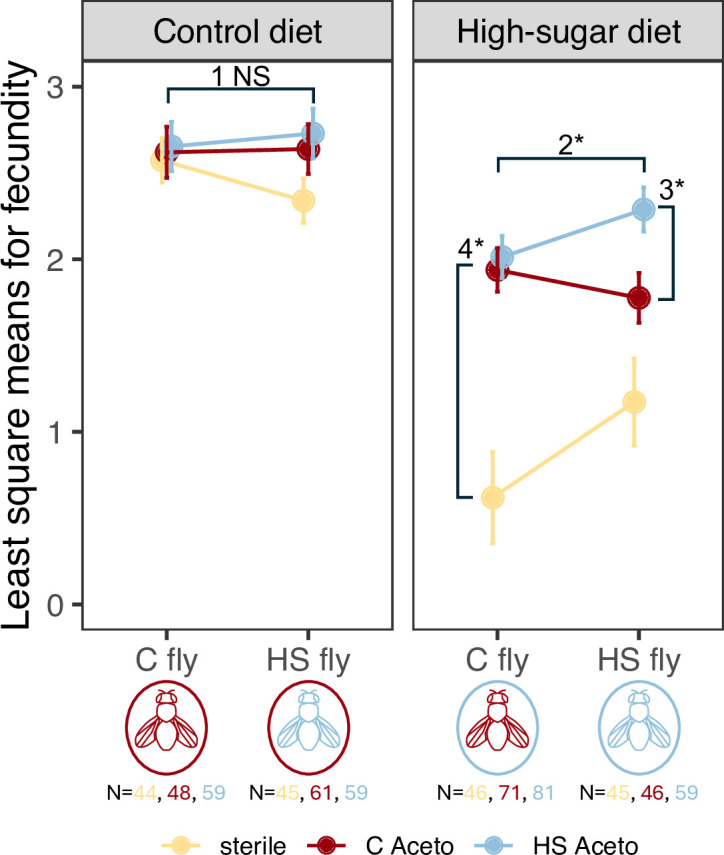
Effects of the *Acetobacter* transplant on fecundity. Least square means (LSMs) from fecundity, modelled with a zero-inflated Poisson distribution show fly genotype × *Acetobacter* × diet interactions shaped fecundity. Plots are faceted by fly diet, where colours represent *Acetobacter* treatment, and the number of age-matched females assayed below the fly diagram. Error bars show standard error for LSMs. Bars marked with asterisks denote significant *post hoc* comparisons. Key results are outlined in brief (see electronic supplementary material, table 7 for full details): (i) no effect of microbiome or fly genotype on the C diet. (ii) HS *Acetobacter* has significantly higher fecundity in HS fly compared with C fly. (iii) HS *Acetobacter* has significantly higher fecundity than C *Acetobacter* in the HS fly. (iv) No difference in fecundity between C and HS *Acetobacter* strains for C flies, but both are higher than the sterile treatment.

## 3. Results

### (a) Fly microbiome responds to high-sugar selection

First, we examined if the microbiome differed between C and HS flies following approximately 150 generations of adaptation to the C or HS diets (figure 2*a*). HS flies were dominated by *A. pasteurianus* (86.7% ± 1.8 s.e.) and an unclassified Acetobacteraceae strain (5.1% ± 1.8 s.e.), while C flies were more diverse with *A. xinjiangensis* (37.3% ± 5.3 s.e.), *A. persici* (16.9% ± 2.6 s.e.) and *A. indonesiensis* (14.8% ± 2.1 s.e.). The most differentially abundant bacteria were *A. pasteurianus* and Acetobacteraceae strain for the HS flies, while *A. xinjiangensis* and *A. indonesiensis* were associated with C flies (figure 2*b*, electronic supplementary material, table 2). These bacteria are unique to either C or HS flies and found across individual flies, demonstrating that the HS diet selected for a different fly microbiome, dominated by *A. pasteurianaus*.

To better understand how the HS diet shaped the microbiome, we compared the genomes of the C and HS *Acetobacter* strains. ANI between the strains was 73.68% (electronic supplementary material, table 3). Strains shared a majority of predicted function (>90% for both strains, electronic supplementary material, figure 1). One key difference was that HS *Acetobacter* encodes for several genes that degrade uric acid, but C *Acetobacter* and other dominant bacteria in the C microbiome do not (electronic supplementary material, table 4). Previous research indicated that nutritionally poor diets increase uric acid accumulation in *D. melanogaster* [43,44], suggesting the importance of HS *Acetobacter* contributing to the degradation of uric acid in the HS diet. We confirmed this prediction by testing the ability for the *Acetobacter* strains to degrade uric acid *in vitro*. Only on the HS diet did HS *Acetobacter* reduce the concentration of uric acid by approximately 50% compared with C *Acetobacter* (figure 2*c*, electronic supplementary material, table 5, *post hoc t*‐test, t = 12.057, d.f. = 7.97, *p* = 2.127 x 10^-6^).

### (b) G_HOST_ × G_MICRO_ × E interactions influence host fecundity

To test if transferring the microbiome could transfer adaptive phenotypes to naive hosts, we performed a fully reciprocal, host genotype × microbiome × diet transplant experiment. Given the simplicity of the microbiome associated with HS flies, we performed the transplant using the *Acetobacter* strains identified as differentially abundant between C and HS flies: *A. indonesiensis* (C *Acetobacter*) and *A. pasteurianus* (HS *Acetobacter*). While not the complete bacterial community from the C flies, we reasoned that because both community complexity (i.e. single dominant species in the HS microbiome versus several in the C microbiome) and phylogenetic diversity (i.e. single dominant *Acetobacter* in the HS microbiome versus two *Acetobacter* species, *Algoriella*, *Commensalibacter*, etc. in the C microbiome) differ, using only C *Acetobacter* still provides an ecologically relevant substitute, while controlling for differences in diversity between the C and HS microbiomes. We then measured fecundity as a higher-order, fitness-associated trait that reflects cumulative response to the multitude of selective pressures.

Fecundity responded to interactions between fly genotype, *Acetobacter* and diet (figure 3, electronic supplementary material, table 6 for summary statistics, electronic supplementary material, table 7 for *post hoc* comparisons). Diet and microbiome explained substantial variation, but the best model fit that explained the most variance included the three-way G_HOST_ × G_MICRO_ × E interaction (electronic supplementary material, figure 4). On average, treatments were more fecund on the C diet, while treatments on HS diets laid 54.3% fewer eggs (C avg. 13.0 eggs ± 0.6 s.e., HS, avg. 5.9 eggs ± 0.4 s.e.). Sterile treatments substantially lowered fecundity, only on the HS diet (+microbes avg. 7.45 eggs ± 0.5 s.e., sterile avg. 1.67 eggs ± 0.4 s.e.). Notably, the response to *Acetobacter* treatment depended on fly genotype for the HS diet. On the HS diet, HS flies with HS *Acetobacter* (avg. 8.4 eggs ± 1.4 s.e.) were almost twice as fecund as flies with the C *Acetobacter* (avg. 4.5 eggs ± 1.0 s.e.). The interaction between fly genotype, microbiome and diet significantly affected fecundity (interaction χ^2^ = 16.4, d.f. = 2, *p* = 0.0003).

## 4. Discussion

Many have predicted that the microbiome could accelerate local adaptation in the host, facilitating rapid phenotypic changes to buffer environmental stressors [6,7,45,46]. This model of adaptation suggests that transplanting locally adapted microbiomes should confer adaptive phenotypes to naive individuals, analogous to transferring beneficial alleles but operating on much faster timescales. While genetic adaptation typically requires many host generations, microbiome-mediated adaptation could theoretically provide rapid benefits to hosts facing novel environmental challenges. However, the success of such microbial benefits likely depend on complex interactions between host genotype and environment. These G_HOST_ × G_MICRO_ × E interactions can either facilitate or impede evolution in challenging environments [47,48], which may explain the variable success of microbiome transplants observed across natural and laboratory settings. The context-dependent nature of these benefits likely reflects the host’s evolved capacity to interact with specific microbes as host genetic architecture fundamentally shapes the ability to capitalize on microbial adaptations. By combining experimental evolution with reciprocal transplants, we tested how ecological context (diet) and evolutionary context (fly genotype) shape the adaptive potential of the microbiome. Our results demonstrate that G_HOST_ × G_MICRO_ × E interactions modify fecundity. Specifically, the HS *Acetobacter* genome encoded uric acid degradation genes and reduced uric acid levels in the HS diet, while the C *Acetobacter* did not. Critically, only fecundity in HS flies benefited from the HS *Acetobacter* under HS conditions, suggesting that host evolutionary history shapes the capacity to leverage microbial adaptations. These findings reveal how selection pressures acting on both hosts and microbes influence the success of microbiome-mediated adaptation and highlight the importance of considering evolutionary history when designing microbiome interventions.

### (a) *Acetobacter* dominates high-sugar microbiome

The HS microbiome was dominated by a single species of bacteria, *A. pasteurianus* (figure 2). *Acetobacter* species are common in flies, both in laboratory settings and wild environments [49–54]. While *Acetobacter* are typically sugar specialists [55], one major axis of variation separating the C and HS microbiomes was the ability to degrade uric acid. In the context of *Drosophila* biology, uric acid accumulation is associated with HS diets in flies [44]. The accumulation of uric acid may also exacerbate the deleterious effects of a HS diet, altering oxidative stress and inflammation that can promote obesogenic phenotypes [56,57]. Uric acid is normally excreted by flies, but high concentrations slow development [58] and shorten lifespans [43,44]. Not all *Acetobacter* species can degrade uric acid, including the C *Acetobacter*, but the ability to degrade is more common in fly-environment than in other free-living *Acetobacter* species [57]. Here, comparative analyses between the C and HS *Acetobacter* genomes showed that HS *Acetobacter* encoded for several genes in the uric acid degradation pathway, while the C *Acetobacter* genome did not. Uric acid degradation may be the key fitness benefit of the HS *Acetobacter* over the C *Acetobacter* in the HS diet.

Microbially mediated uric acid degradation represents a fascinating case of coupled fitness effects: direct benefits for HS *Acetobacter* and indirect advantages for their host flies. During experimental evolution, exposure to the HS diet created strong selection pressures on both organisms. The physiological stress of HS consumption generates excess uric acid in flies, creating both a challenge for the host and a novel metabolic opportunity for bacteria. The emergence of *A. pasteurianus* as the dominant species reflects its competitive advantage—*Acetobacter* species capable of degrading uric acid consistently outcompete those that cannot in fly environments [57]. This metabolic capability likely explains both its persistence in the stressful HS diet and its dominance in the gut community. The relationship proved beneficial for both partners: while flies have limited capacity to manage uric acid on their own, primarily relying on decreased uptake or increased excretion [58–60], they could now exploit their microbiome’s novel metabolic capability. Microbially mediated uric acid detoxification represents a fascinating case of coupled benefits, with direct metabolic advantages for the bacteria and protection from toxicity for the host. This detoxification service may be particularly valuable given that traditional host mechanisms of uric acid control become overwhelmed under sustained dietary stress. Beyond flies, microbially encoded ureases (as we observed in HS *Acetobacter*, electronic supplementary material, table 4) play crucial roles across diverse host–microbe systems. These pathways not only detoxify metabolic by-products but also mitigate limited nutrient availability by accessing new protein sources for a wide range of hosts, from ruminants [61] to hibernating squirrels [62] to mice [63] to infant humans [64]. This convergence across such diverse systems suggests that metabolic detoxification and nutrient processing may be a common mechanism underlying beneficial host–microbe interactions. However, more work is necessary to understand the relationship between mitigating limited nutrients and detoxifying dietary stressors in host–microbiome interactions.

This metabolic complementation exemplifies a broader pattern where microbiomes can provide their hosts with novel functions that expand adaptive potential in stressful environments [6,9,10]. Unlike traditional evolutionary responses that require genetic changes in the host, microbial adaptation can rapidly provide new metabolic capabilities through shifts in community composition. The success of this strategy in our experimental system suggests that hosts may commonly exploit microbial metabolic diversity to cope with environmental challenges, particularly when facing novel stressors that exceed their intrinsic physiological capabilities.

### (b) Adaptive potential of the microbiome depends on evolutionary and ecological context

To shape adaptive potential, the microbiome must contribute to host phenotypic variation, not unlike traditional additive genetic variance [6]. The G_HOST_ × G_MICRO_ × E interaction illustrates how diet (the ecological context), when matched with host genotype (the evolutionary context), may increase the fitness benefit of locally adaptive microbiomes (figure 3), like for fecundity increase for HS flies with HS *Acetobacter* on the HS diet. Alternatively, mismatches in the G_HOST_ × G_MICRO_ × E interaction may impede the transfer of adaptive potential. The naive host, C flies, did not receive a fecundity increase from the HS *Acetobacter* in the HS diet (figure 3).

The G_HOST_ × G_MICRO_ × E interactions we observed in fecundity highlight how microbiomes can shape host evolution, while simultaneously revealing the constraints on these interactions. Because host genotypes may vary in their ability to leverage beneficial microbes in stressful environments, genetic interactions may limit the benefits of locally adaptive microbiomes. Understanding the evolutionary dynamics of these G_HOST_ × G_MICRO_ × E interactions requires tracking the temporal sequence of adaptation in both partners. Diet frequently restructures the microbiome in *Drosophila* [14], and in our system, likely through both direct effects of high sucrose and indirect effects via fly production of uric acid. Intriguingly, another experimental evolution study by Martino *et al*. showed that *Lactobacillus* evolved growth-promoting mutations in a low-nutrition diet in just two fly generations [16]. More so, the growth-promoting mutations in *Lactobacillus* evolved in response to the low-nutrition diet alone—independently of any fly selection—but the ability of the evolved *Lactobacillus* to increase growth in the poor diet also helped improve larval fly growth. While our study similarly demonstrates indirect benefits of bacterial adaptation, the significant G_HOST_ × G_MICRO_ × E interaction suggests an additional layer of complexity—HS flies evolved mechanisms to better capitalize on the benefits of HS *Acetobacter* (figure 3). Several factors may explain these different outcomes. First, Martino *et al*. [16] only lasted 20 generations, while our study spans approximately 150 generations of fly adaptation to the HS diet. Second, *Lactobacillus* and *Acetobacter* have divergent effects on many aspects of *Drosophila* physiology [65,66]. Third, different diets create distinct selective pressures that lead to different evolutionary outcomes, including the potential for reduced benefits derived from the microbiome [67]. This complexity highlights how diet and microbial variation together shape the evolvability of host–microbiome interactions [68]. While more work is needed to untangle the different drivers of host–microbiome evolution, particularly through longitudinal sampling of evolutionary trajectories, our results clearly demonstrate how both ecological context and host evolution interact to shape the adaptive potential of the microbiome.

### (c) Research priorities

Experimental evolution has provided fundamental insights into adaptation, yet traditional E&R approaches have illuminated many important drivers of adaptation [20,69] while largely overlooking the microbiome’s role. This oversight is particularly striking given that a recent survey of 10 E&R experiments revealed that microbiomes frequently responded to experimental evolution, especially in metabolism-related traits [19]. Our study addresses this gap by explicitly incorporating microbiome dynamics into the E&R framework. Through our reciprocal transplant experiment, we uncovered how the microbiome contributes to host adaptation—effects previously unseen when focusing on the *Drosophila* genome alone. This work complements recent findings in *Drosophila* showing that microbial variation can drive rapid phenotypic and genetic change in semi-natural populations [65,66,70]. By simultaneously comparing both host and microbial adaptation, our study provides the first comprehensive demonstration of their intertwined adaptive trajectories in an E&R framework.

G_HOST_ × G_MICRO_ × E interactions revealed unexpected complexity in adaptation to the HS diet. Most notably, the ability of HS *Acetobacter* to degrade uric acid exemplifies diffuse coevolution [71]. We propose a model where the HS diet triggered increased uric acid production by flies, creating a new niche for *Acetobacter* capable of exploiting this metabolite. The subsequent expansion of these bacteria then reduced toxicity for their hosts, generating a positive feedback loop between host and microbe fitness. This pattern suggests that in many systems, host and microbiome evolution may be fundamentally interconnected through such feedback mechanisms.

There are several limitations to our study. First, the fly microbiome is a dynamic system that reflects the acquisition and loss of different bacteria [72,73]. We only examined the microbiome at the end of experimental evolution, as is typically done in traditional E&R experiments [69]; how quickly the microbiome changed over the course of fly adaptation to the HS diet is unknown. A key research priority is to track the joint evolutionary trajectory of the host and microbiome as flies adapt to a stressful environment. Many other host systems show substantial fluctuations in microbiome composition over time [74–76], and thus systems like *Drosophila* offer great promise in understanding how dynamic microbiomes can contribute to host adaptation. Second, we focused on fecundity at a single time point in 8−10 day old flies as the key fitness-associated trait. However, different aspects of life history, like development or fecundity over the host lifespan, also contribute to fitness. The relative importance of the microbiome may vary across these different fitness components [17,70,77]; building a more comprehensive map of host–microbiome interactions underlying fitness is an important research priority. Third, we focused on uric acid degradation because of its importance in mitigating HS stress in *Drosophila* [43,44] as well as its emerging importance in other host–microbiome interactions [62–64]. While the C and HS *Acetobacter* strains encoded a majority of the same functions, they also differed across a wide range of functions (electronic supplementary material, figure 1). More extensive screening of other functions that differentiate the two *Acetobacter* strains will provide deeper insight into how microbes shape host adaptation.

Looking forward, understanding these G_HOST_ × G_MICRO_ interactions will be crucial for engineering beneficial microbiomes to address pressing problems in public health and agriculture [6,47,48,78]. The success of microbiome-based interventions depends not only on identifying beneficial microbes but also on understanding how host genetic variation modulates their effects. Our results demonstrate that host genotype can fundamentally alter the benefits derived from microbial adaptations—a critical consideration for translating microbiome research into effective treatments. Future work utilizing experimental evolution should focus on elucidating how the joint evolution of host and microbiome shapes adaptive outcomes, particularly across different environmental contexts. Understanding these G_HOST_ × G_MICRO_ × E interactions may be especially important given the increasing recognition that diet, host genetics and microbiome variation all contribute to phenotypic outcomes. Such research will reveal fundamental principles about how natural selection acts on host–microbe systems, while informing practical applications in medicine and agriculture.

## Data Availability

Sequences were deposited in NCBI SRA under BioProjects PRJNA1190096 for C Acetobacter genome, PRJNA1190097 for HS Acetobacter genome and PRJNA1209086 for 16S rRNA microbiome profiling. Phenotypic data was deposited in Dryad [79]. Code is available on Zenodo [80]. Supplementary material is available online [81].
